# Hypoalbuminemia in children with acute lymphoblastic leukemia: relation to asparaginase therapy and impact on high dose methotrexate elimination

**DOI:** 10.1007/s00280-024-04713-0

**Published:** 2024-09-21

**Authors:** Sophie Rex Christensen, Christina Friis Jensen, Jesper Heldrup, Zachary Taylor, Laura B. Ramsey, Steen Rosthøj

**Affiliations:** 1 Section of Pediatric Hematology and Oncology, Department of Pediatrics and Adolescence Medicine, Aalborg University Hospital, Reberbansgade, 9000 Aalborg, Denmark; 2 Childhood Cancer and Research Unit, University Children’s Hospital, Lund, Sweden; 3 Division of Translational and Clinical Pharmacology, Cincinnati Children’s Hospital Medical Center, Cincinnati, OH USA; 4 Division of Clinical Pharmacology, Toxicology & Therapeutic Innovation, Children’s Mercy Kansas City, Kansas City, MO USA

**Keywords:** Childhood leukemia, Asparaginase, Hypoalbuminemia, Methotrexate toxicity/clearance

## Abstract

**Purpose:**

High-dose methotrexate (HDMTX) therapy is an important component in treatment regimens for acute lymphoblastic leukemia (ALL). Courses are associated with a risk of renal injury, delayed elimination, and increased systemic toxicity. Recently hypoalbuminemia has been recognized as yet another risk factor.

**Methods:**

To examine the impact of serum albumin we reviewed 325 HDMTX 5 g/m2 courses in a cohort of 51 children treated on the NOPHO ALL 2008 protocol, dividing the courses into four groups with different levels of baseline albumin (A < 25 g/L, B 25–29 g/L, C 30–34 g/L and D ≥ 35 g/L).

**Results:**

Hypoalbuminemia was present in 51% of the courses, mostly in the early phases of chemotherapy while asparaginase therapy is ongoing, and especially if given less than 2 weeks after a dose (78%). Hypoalbuminemia had a significant impact on the end-of-infusion serum MTX, depending on the degree of hypoalbuminemia: MTX > 150 µM was seen in 37%, 32%, 20% and 8% in groups A to D. Serum albumin < 30 g/L was significantly associated with low MTX clearance < 10 L/h/1.73m2 (78% vs. 36%) and high AUC ≥ 1000 µM*h (44% vs. 31%). The frequency of rising creatinine or prolonged elimination was not increased, but the risk of stomatitis was significantly higher (42% vs. 19%).

**Conclusion:**

Low serum albumin is caused by concurrent asparaginase therapy and has a clinically significant impact on MTX disposition. Guidelines for administering HDMTX may need adjustment if serum albumin < 30 g/L, and, if possible, HDMTX courses should not be scheduled soon after asparaginase doses.

**Supplementary Information:**

The online version contains supplementary material available at 10.1007/s00280-024-04713-0.

## Introduction

High-dose treatment with the folate antagonist methotrexate (MTX) was shown to be feasible in the 70’es [1, 2], and since then high-dose MTX (HDMTX) courses have become an important component in treatment regimens for several malignancies: leukemia, lymphoma, osteosarcoma, CNS tumors. In childhood acute lymphoblastic leukemia (ALL) HDMTX therapy has permitted omission of prophylactic CNS irradiation [3, 4] and has contributed to improved results, with cure rates now approaching 85–90% [5]. High-dose infusions are still associated with considerable risk of systemic toxicity, however, and courses need to be administered with pharmacokinetic monitoring and meticulous attention to folinic acid rescue, hydration, urine output, and alkalinization of urine. Delayed MTX elimination (DME) may occur because of acute renal dysfunction caused by renal hypoperfusion, reduced glomerular function, or tubular MTX precipitation. A significant increase in serum creatinine occurs in up to 20% of courses [6], with a 50% rise within 36 h of starting the infusion as a marker of DME [7, 8]. In case of renal failure and very high MTX concentrations treatment with glucarpidase, cleaving MTX, should be used [9].

Renal impairment and DME with increased risk of systemic toxicity (mucositis, bone marrow suppression) often occurs unpredictably and unexpectedly. Several risk factors are known: older age, male sex, MTX dosage and schedule, vomiting or inadequate urine output during the infusion, intercurrent infection, or concomitant use of nephrotoxic or interacting drugs [10]. Polymorphisms in genes involved in drug disposition, especially SLCO1B1, may play a role [11–14]. Recently it has been recognized that hypoalbuminemia when starting the infusion also is a risk factor [15, 16]. Further studies have confirmed the importance in adults [17–19]. The importance in children has not yet been elucidated. Hypoalbuminemia was not included in a recent list of risk factors [20], but early reports have now appeared [21, 22].

Hypoalbuminemia is often present when starting HDMTX infusions in children with ALL, probably as a side effect of asparaginase (ASP), used in the treatment of ALL since the 1960’es [23], which causes depletion of L-asparagine, “starving” lymphoblasts but also reducing hepatic protein synthesis [24]; pegylation of ASP prolongs this effect [25]. Aiming to estimate the frequency of hypoalbuminemia in HDMTX courses and to describe the impact of different degrees of hypoalbuminemia on renal toxicity, elimination of MTX, and systemic toxicity, we decided to review HDMTX courses given to a cohort of children with ALL diagnosed and treated at our institution. In addition, we wanted to explore the relation of reduced serum albumin to preceding doses of ASP.

## Materials and methods

From July 2008 through December 2018, a consecutive series of 51 non-Down children and adolescents aged 1–17 years were diagnosed with ALL and included in the NOPHO ALL 2008 protocol at our institution. [5] The cohort comprised 25 boys and 26 girls; 44 children aged 1–14 years and 7 adolescents aged 15–17 years; and 47 with pre-B ALL and 4 with T-ALL. Patients were stratified into standard risk (SR n = 22), intermediate risk (IR n = 19), and high-risk (HR n = 10) treatment groups as determined by phenotype, WBC at diagnosis, CNS involvement, cytogenetic findings and minimal residual disease response after 29 days. Patients with SR and IR were to receive three HDMTX 5 g/m2 courses at 3-week intervals during consolidation and five courses at 8-week intervals after starting maintenance therapy. Patients with HR ALL were to receive six HDMTX courses as part of nine chemotherapy blocks given at 3–4-week intervals, and three courses at 24-week intervals after starting maintenance therapy. Collectively, a total of 329 HDMTX courses were given: 150 to SR patients, 138 to IR patients, and 41 to HR patients.

## HDMTX treatment protocol

HDMTX was administered according to protocol guidelines. Initiation required normal creatinine, absolute neutrophil count (ANC) ≥ 0.5 × 109/L, platelet count > 50 × 109/L, alanine aminotransferases (ALAT) < 10 × ULN, and urinary pH ≥ 7. After pre-hydration for 4 h, MTX 5000 mg/ m2 was infused over 24 h, giving 10% of the dose in the first hour; intrathecal MTX was administered during the infusion. Hydration with fluids containing bicarbonate was given at a rate of 3000 ml/ m2/24 h, increased to 4500 ml/ m2/24 h in case of a 50% rise in creatinine or very high MTX concentrations (> 250 µM after 23 h, > 30 µM after 36 h, or > 10 µM after 42 h). Urine output and urine pH (target 7.0 to 7.5) was monitored throughout the cycle. Folinic acid rescue 15 mg/m2 i.v. was given after 42 and 48 h and continued every 6 h until MTX was eliminated (MTX ≤ 0,2 µM), with dose increments according to a sliding scale for MTX > 1.0 µM. Use of glucarpidase was to be considered if MTX > 30 µM after 36 h or > 10 µM after 42 h, or if high concentrations were combined with a creatinine rise > 2 × ULN.

Nephrotoxic drugs or drugs potentially interacting with MTX-excretion were to be avoided. Pneumocystis prophylaxis with sulfamethoxazole/trimethoprim was withheld during the week with HDMTX.

Except for some courses given to HR patients, oral 6-mercaptopurine 25–100 mg/ m2 was co-administered with HDMTX. For courses given during the first 33 weeks of ALL chemotherapy, parallel therapy with pegylated asparaginase (PEG-ASP) doses 1000 U/m2 intra muscular was ongoing. SR and IR patients were randomized to either 15 doses at two-week intervals or eight doses with six-week intervals between the last three) [26], and HR patients received nine doses at 3–4-week intervals.

## Data collection and analysis

Patient records were reviewed to describe the course of the 329 HDMTX infusions. For each course the following data were extracted: pre-infusion blood counts and biochemistry (albumin, creatinine and estimated GFR); MTX concentrations and biochemistry during and after the infusion until MTX was eliminated (i.e. serum MTX ≤ 0,2 µM); and time from start of infusion to elimination of MTX. Time since the last scheduled dose of PEG-ASP was determined. For the 3 weeks following discharge systemic toxicity was recorded by noting occurrence of clinically verified stomatitis (any grade) and nadir values of ANC and platelet counts.

To explore the impact of albumin level on the course of therapy, infusions were divided into four groups with different levels of serum albumin measured 4–24 h prior to starting the infusion: group A < 25 g/L, B 25–29 g/L, C 30–34 g/L, and D ≥ 35 g/L. MTX accumulation and elimination, creatinine changes, and systemic post-infusion toxicity in the four groups were compared descriptively, looking for trends related to the albumin level. In an overall analysis, the frequencies of therapy-related events were compared in courses with pre-infusion serum albumin < 30 g/L (group AB) and ≥ 30 g/L (group CD).

The pharmacokinetic data from the 329 courses of HDMTX were analyzed by non-linear mixed effects modeling performed in NONMEM (version 7.2.0, ICON, Ellicott City, Maryland, USA). A previously published population pharmacokinetic model for HDMTX in Nordic pediatric patients with ALL was loaded into NONMEM to analyze these data [27]. In brief, this model comprises a 3-compartment structural model that includes a patient’s body surface area, baseline serum creatinine, and time-varying serum creatinine to describe the pharmacokinetics of HDMTX and generate patient-specific pharmacokinetic MTX parameters (e.g., central, and peripheral volumes of distribution, clearance, area under curve (AUC)).

## Statistics

The study is exploratory without prior hypotheses for statistical testing. Averages, median values, counts, and percentages are presented and compared descriptively. Some apparent associations have been evaluated by calculating odds ratios (OR) with 95% confidence limits. Pharmacokinetic data were tested across the albumin groups using a one-way ANOVA. Following a statistically significant p value, a Dunnett’s test was performed to compare each group to the D ≥ 35 g/L reference group.

The study is retrospective and descriptive, without any prior hypotheses for statistical testing. Averages, median values, counts, and percentages are presented and compared descriptively. To generate appropriate hypotheses for future research, apparent associations between hypoalbuminemia and adverse events have been evaluated by calculating univariate, unadjusted odds ratios (OR) with 95% confidence limits. The pharmacokinetic data were tested across the albumin groups using a one-way ANOVA; Following a statistically significant p value, a Dunnett’s test was performed to compare each group to the D ≥ 35 g/L reference group.

## Ethics and statement of human and animal rights

The study was retrospective and non-interventional, and collected data was anonymized. The study was registered and approved by North Jutland regional authorities. Informed consent was not required.

## Results

**Fig. 1:**
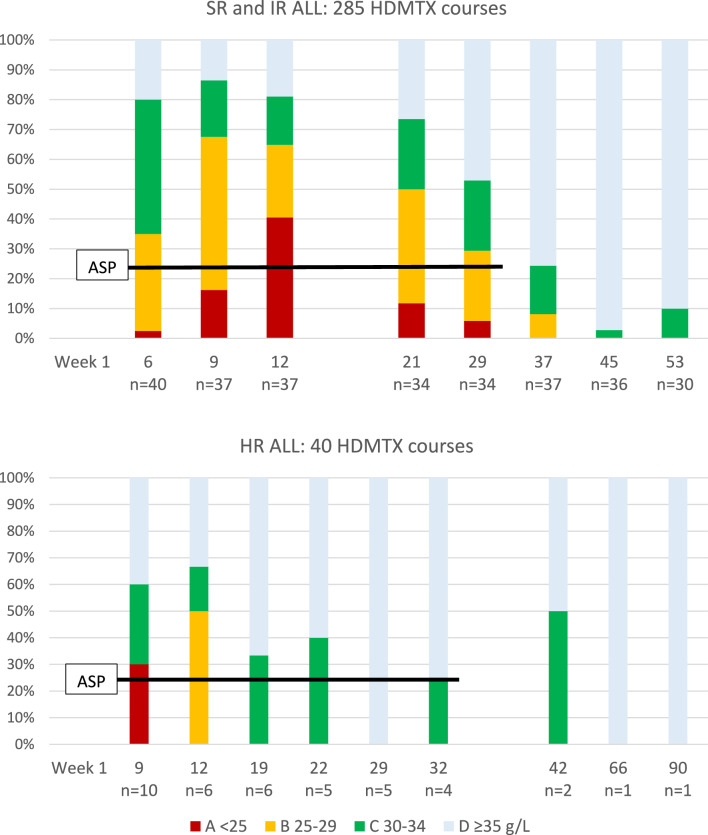
Hypoalbuminemia in sequential HDMTX courses. The occurrence of hypoalbuminemia of different degrees (< 25 g/L, 25–29 g/L, or 30–34 g/L) at the time of HDMTX infusions during NOPHO 2008 chemotherapy for ALL. Upper figure: SR and IR patients (n = 41). The first three courses were given during consolidation therapy, the last five after starting maintenance therapy. PEG-asparaginase doses (ASP) were given i.m. from week 5 to week 33, in most cases at 2-week intervals (black bar). Lower figure: HR patients (n = 10). The first six courses were given as part of nine chemotherapy blocks at 3–4-week intervals, the last three during maintenance therapy. ASP was given week 5 and every 3–4 weeks from week 10 to 33. All time points are as scheduled by protocol; some HDMTX courses were delayed for various clinical reasons

A pre-infusion serum albumin was measured in 325 courses. Albumin was below normal in 165 (group A 31, group B 68, group C 66) and normal in 160 courses (group D). Hypoalbuminemia was a little more frequent in children aged > 5 years (59% of courses vs. 48%). In SR and IR patients, serum albumin was low in > 75% of the three HDM courses given in consolidation phase, and in > 50% of the first two courses in the later maintenance phase (Fig. 1A). In HR patients, hypoalbuminemia was less frequent and less severe (Fig. 1B).

## Relation of hypoalbuminemia to asparaginase therapy

**Fig. 2:**
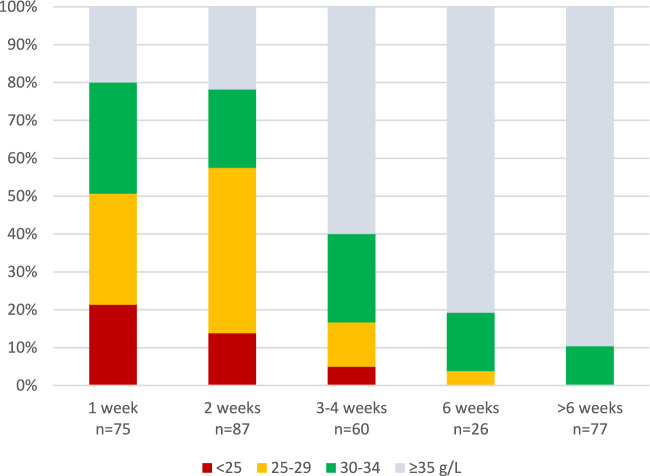
Relation of hypoalbuminemia to time since the last dose of PEG-asparaginase. Infusions given to all three risk groups of ALL are included (n = 325). Time since last dose has been determined as per protocol schedule; some courses have been delayed by a few days relative to the asparaginase dose

Hypoalbuminemia coincided with parallel, ongoing asparaginase therapy. After cessation of asparaginase, serum albumin was rarely and only slightly reduced (Fig. 1). The albumin level depended on the time since the last dose of asparaginase, being often moderately or severely suppressed 1–2 weeks after a dose and only occasionally and mildly suppressed more than 4 weeks after a dose (Fig. 2).

**Fig. 3:**
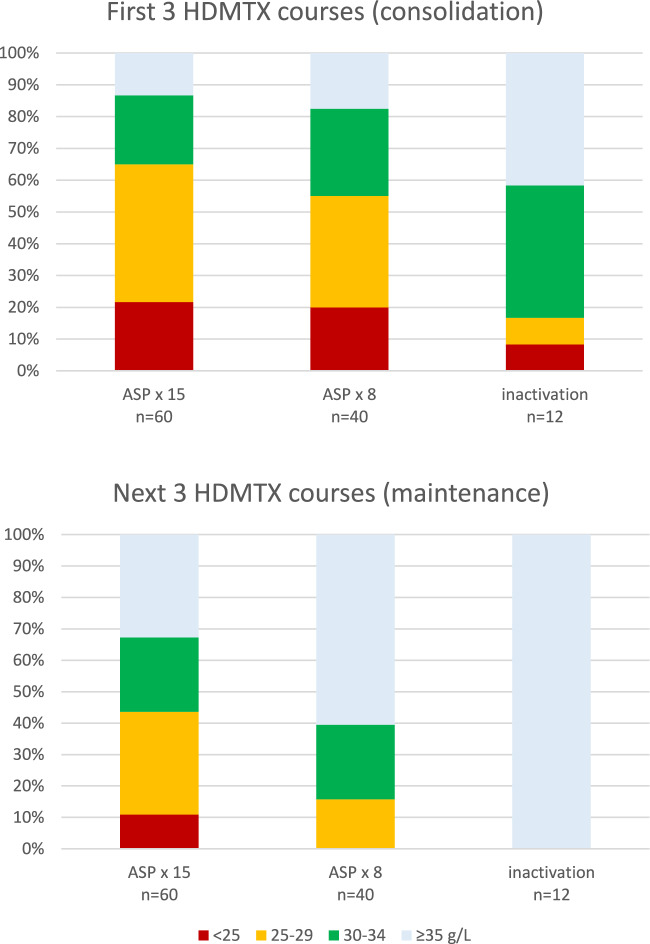
Hypoalbuminemia in relation to intensity of asparaginase therapy. Comparison of serum albumin levels in 38 children with SR and IR ALL treated with 15 doses of ASP at 2-week intervals (ASP × 15, 20 cases), 5 doses at 2-week intervals followed by 3 doses at 6-week intervals (ASP × 8, 14 cases), and children with demonstrated inactivation of ASP (4 cases). The upper panel shows the first three HDMTX courses given during consolidation therapy, the lower panel the next three courses given after starting maintenance chemotherapy, while ASP therapy is still ongoing

SR and IR patients received asparaginase either 15 doses at 2-week intervals or 5 doses at 2-week intervals during consolidation followed by 3 doses 6-weekly. The frequency and severity of hypoalbuminemia in the two groups was similar in the consolidation phase, but during maintenance chemotherapy the latter group had less frequent and severe albumin suppression (Fig. 3). In four cases drug monitoring demonstrated inactivation of asparaginase; these children had less pronounced suppression during consolidation and normal serum albumin during maintenance.

## Impact of hypoalbuminemia on the course of HDMTX

**Fig. 4:**
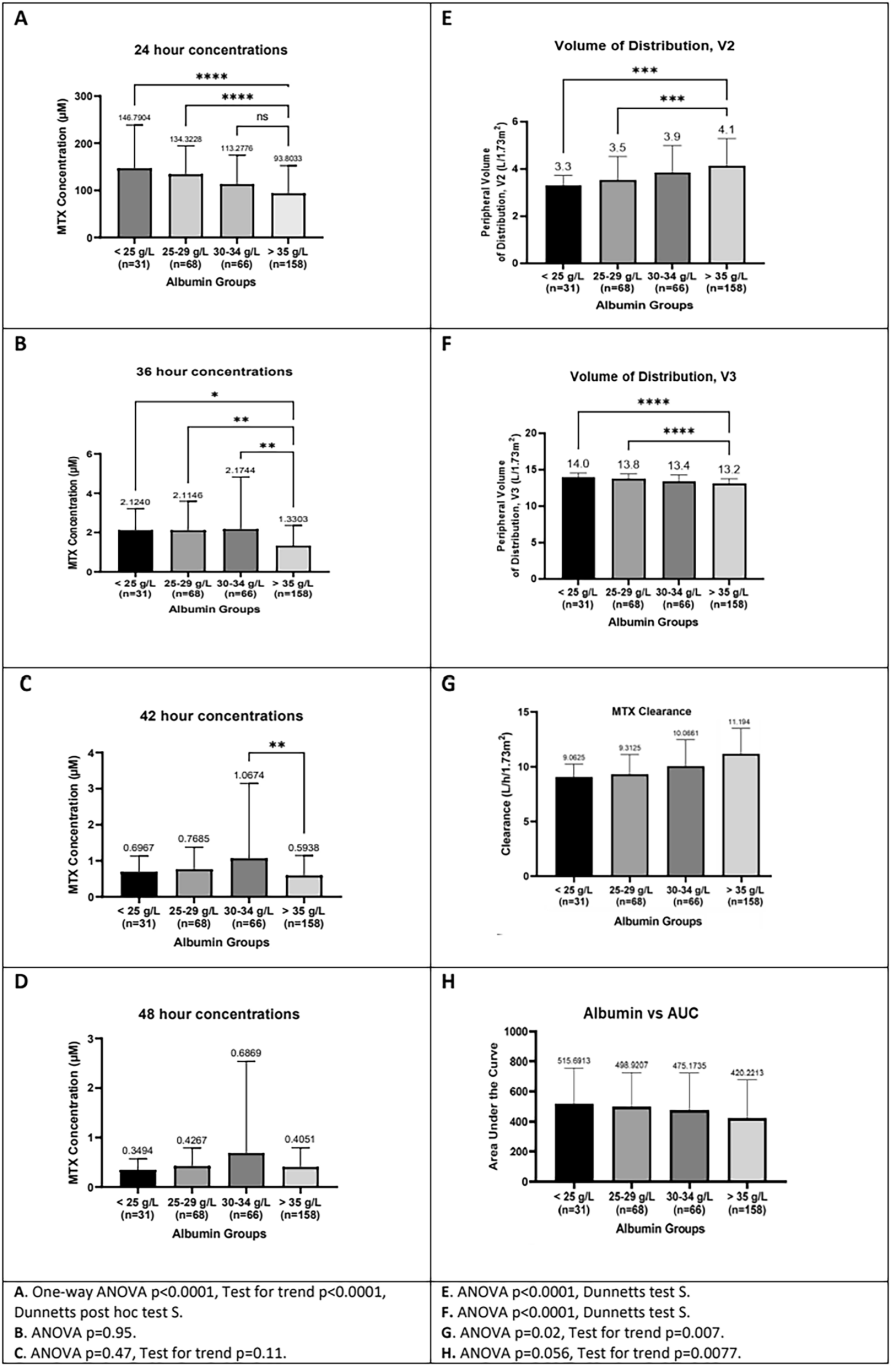
Pharmacokinetic analysis of serum MTX elimination. Data for HDMTX courses with different levels of baseline serum albumin (< 25 g/L, 25–29 g/L, 30–35 g/L, ≥ 35 g/L) have been compared. Panel** A-D:** MTX concentrations at and after end of infusion; an outlier treated with glucarpidase because of very high MTX concentrations is present in group C. Panel **E–H:** Peripheral volumes of distribution, MTX clearance, and AUC have been determined using a three-compartment pharmacokinetic model (MTXPK.org) loaded into NONMEM

MTX concentrations, elimination, pharmacokinetics, and toxicity in the four groups with different albumin levels are compared in Table 1. The pharmacokinetic analyses are shown graphically in Fig. 4.

**Table 1:** Comparison of methotrexate elimination and toxicity after 325 high-dose infusions in four groups with different pre-infusion serum albumin levels. For serum-MTX median values are given, all other values are averages or percentages

	Group A	Group B	Group C	Group D
	< 25 g/L	25–29 g/L	30–34 g/L	≥ 35 g/L
	n = 31	n = 68	n = 66	n = 160
MTX-h23 µM	127,6	129,3	97,5	78,6
MTX-h36 µM	1,83	1,73	1,28	1,04
MTX-h42 µM	0,63	0,57	0,53	0,42
MTX-h48 µM	0,27	0,28	0,29	0,30
MTX-h23 ≥ 250 µM	3%	4%	3%	1%
MTX-h23 ≥ 150 µM	37%	32%	20%	8%
MTX-h36 ≥ 3,0 µM	20%	22%	15%	6%
MTX-h42 ≥ 1,0 µM	16%	26%	18%	14%
MTX-h48 > 0,2 µM	77%	69%	71%	69%
Elimination time hours	57,3	59,4	62,6	60,8
Elimination time > 72 h	3%	12%	11%	12%
Elimination time > 96 h	0%	3%	6%	3%
Creatinine pre-infusion µM	39,5	37,9	32,9	32,3
eGFR-h00 ml/min/1.73 m^2^	135	130	149	147
eGFR-h23 ml/min/1.73 m^2^	131	139	145	144
eGFR-h36 ml/min/1.73 m^2^	146	147	145	153
eGFR 42 ml/min/1.73 m^2^	153	153	155	155
Creatinine rise > 50%	3%	7%	17%	11%
Creatinine rise > 25 µM	3%	1%	5%	1%
Central compartment V1 L/1.73 m2*	(16,5)	(16,5)	(16,5)	(16,5)
Peripheral compartment V2 L/1.73 m^2^	3,3	3,5	3,9	4,1
Peripheral compartment V3 L/1.73 m^2^	14,0	13,8	13,4	13,2
Clearance L/h/1.73 m^2^	9,06	9,30	10,08	11,16
Clearance < 10 L/h/1.73 m^2^	81%	76%	47%	32%
AUC µM*h	1135	1098	1045	924
AUC ≥ 1000 µM*h	45%	44%	38%	28%
PLC nadir × 10^9^/L	107	74	94	101
PLC nadir < 50 × 10^9^/L	45%	51%	45%	38%
ANC nadir × 10^9^/L	0,51	0,66	0,61	0,78
ANC nadir < 0,5 × 10^9^/L	52%	49%	42%	38%
Stomatitis	35%	46%	21%	18%

*The model did not include an inter-individual variability value for central volume, giving V1 the same value in all groups

The end-of-infusion serum MTX concentrations were higher in the hypoalbuminemic groups. The elevation was most pronounced in groups A and B, where the median steady-state concentration was more than 125 µM and more than 30% had high values > 150 µM. The concentration differences disappeared during the first 24 h of post-infusion hydration. The average time to elimination (serum MTX ≤ 0.2 µM) was not increased (close to 60 h in all groups), and prolonged elimination > 96 h was not more frequent.

In groups A and B, serum creatinine was somewhat higher and eGFR lower when starting the course. Catch-up in eGFR occurred at h36, and at h42 mean values were > 150 ml/min/1.73 m2 in all groups (Figure S1). Renal toxicity (creatinine rise > 50%) was less frequent in groups A and B, occurring at rates below 10%. One case in group C had very high MTX-h36 (66.5 µM) and a pronounced creatinine rise from 60 µM to 206 µM; a single dose of glucarpidase was given, renal function recovered, but elimination lasted 12 days.

## Pharmacokinetics

The model did not estimate the inter-individual variability for the V1 parameter; all individual values were estimated to 16.5 L/1.73m2. With decreasing serum albumin there was a pronounced decrease in V2 (a vascular tissue space) accompanied by a moderate increase in V3 (a less vascular tissue space). MTX clearance was slower in the hypoalbuminemic groups, being < 10L/h/1.73 m2 in more than 75% of group A and B (Figure S2). AUC correspondingly was higher, exceeding 1000 µM*h in almost half of group A and B courses.

## Systemic toxicity

The toxicity following courses was somewhat increased. Stomatitis occurred in more than one third in groups A and B. Myelotoxicity was slightly more pronounced in these groups, a neutrophil nadir < 0.5 × 109/L and a platelet nadir < 50 × 109/L both occurring in about 50%.

## Definition of clinically significant hypoalbuminemia

The differences between group C and group D were relatively modest. Groups AB and CD are compared in Table 2. Serum albumin < 30 g/L was significantly associated with low MTX clearance, high end-of-infusion concentration, and high MTX exposure. The risk of stomatitis following the course was twice as high.

**Table 2:** Comparison of adverse events in high-dose Methotrexate infusions with serum albumin < 30 g/L (group AB, n = 99) and ≥ 30 g/L (group CD, n = 226). The degree of association has been assessed by Odds Ratios (OR) with 95% confidence intervals (95% CI); lower limit of CI > 1.0 corresponds to statistical significance at level P > 0.05

	Group AB	Group CD		
	< 30 g/L	≥ 30 g/L		
	n = 99	n = 226	OR	95% CI
MTX-h23 ≥ 250 µM	4%	2%	2.34	0.57–9.54
MTX-h23 ≥ 150 µM	33%	12%	3.85	2.14–6.90
MTX-h36 ≥ 3,0 µM	21%	8%	2.93	1.50–5.75
MTX-h42 ≥ 1,0 µM	23%	15%	1.71	0.95–3.09
MTX-h48 > 0,2 µM	72%	70%	1.09	0.65–1.84
Elimination time > 72h	9%	12%	0.77	0.35–1.71
Elimination time > 96h	2%	4%	0.56	0.12–2.69
Creatinine rise > 50%	6%	12%	0.46	0.18–1.14
Creatinine rise > 25 µM	2%	2%	0.91	0.17–4.78
Clearance < 10 L/h/1.73 m^2^	78%	36%	6.15	3.56–10.61
AUC ≥ 1000 µM*h	44%	31%	1.82	1.12–2.96
PLC nadir < 50 × 10^9^/L	49%	40%	1.48	0.92–2.38
ANC nadir < 0,5 × 10^9^/L	49%	39%	1.54	0.95–2.47
Stomatitis	42%	19%	3.14	1.87–5.27

## Discussion

High-dose infusions of MTX, a folate antagonist blocking intracellular dihydrofolate reductase and thymidylate synthetase, are used widely in the treatment of malignancies but carry a risk of significant toxicity, the causes of which need to be clarified. In this retrospective study of the role of serum albumin in 325 HDMTX 5g/m2 courses given to children with ALL we found that hypoalbuminemia was a frequent occurrence, apparently caused by preceding asparaginase therapy. There was a clear relation to recent doses of ASP and to the intensity of ASP therapy, hypoalbuminemia being less frequent in SR/IR children receiving reduced number of doses and in HR children with longer intervals between doses. A low serum albumin, especially when < 30 g/L, was associated with lower MTX clearance and had a marked impact on end-of-infusion MTX concentrations that were very high in one third of infusions. Creatinine rises did not occur more frequently, and MTX elimination was not delayed. Later systemic toxicity, however, was more pronounced with a doubled risk of stomatitis.

This study is retrospective and therefore subject to several limitations. Clinical information in records may have been inaccurate and incomplete, and some laboratory values were missing. Accurate grading of stomatitis was not possible retrospectively. Information on concurrent infection, concomitant medication and management of fluid balance was not collected. Deviations from protocol guidelines were not registered, so that strict adherence cannot be assumed. Presence of known risk factors for DME could not be recorded systematically. Therefore, the interaction between hypoalbuminemia and other factors could not be elucidated, and multifactorial modelling was not possible. Thus, the study is a single-factor analysis. It is a strength in interpreting the results, however, that the study cohort is composed of children with the same diagnosis receiving HDMTX at the same dose according to a specified guideline. Also, the analytic design with division into four groups permitted exploration of the importance of the degree of hypoalbuminemia. Overall, we think that our data material is sufficiently robust for a descriptive analysis that can provide reliable findings.

Low serum albumin is encountered quite frequently in children treated for cancer. At the time of diagnosis hypoalbuminemia may be present in half the children, and if severe may be associated with reduced survival [28]. Later during chemotherapy, a low serum albumin often can be attributed to well-known complications: malnutrition or hepatotoxicity quite commonly, renal or intestinal loss occasionally. In previous studies in adults, hypoalbuminemia has been reported in connection with 18% to 27% of HDMTX courses [15–19]. In two childhood series from low-middle-income countries 32% and 35% of the children were hypoalbuminemic, possibly because of malnutrition [21, 22].

In the present study the frequency of reduced serum albumin was close to 50%. The cause of this high prevalence is not immediately obvious. We found, however, a clear relation to recent administration of ASP. A single dose of L-asparaginase causes depletion of L-asparagine for two weeks, blocking lymphoblast replication, but also reducing synthesis of various proteins including albumin [24, 25]. ASP therapy in the NOPHO ALL-2008 protocol was intensive and prolonged, most children receiving 15 doses at 2-week intervals after induction. During consolidation hypoalbuminemia was present in up to 85% of HDMTX courses, often with values < 30 g/L. During maintenance, hypoalbuminemia was still frequent in children receiving ASP at 2-week intervals, less frequent and severe in those receiving ASP at 6-week intervals, and absent in those with inactivation of ASP. After cessation of ASP therapy, albumin levels were normal. Thus, it appears that ASP is responsible for the hypoalbuminemia, which is a biomarker of ASP activity, previously reported to influence the clearance of dexamethasone [25].

Hypoalbuminemia may influence the pharmacokinetics of MTX in several ways [19, 29]. MTX is partially bound to albumin in the intravascular compartment. Approximately 70% is cleared by the kidneys, mostly by glomerular filtration, to a smaller extent by tubular secretion. Free MTX is distributed in the extravascular space and taken up by cells. Hypoalbuminemia reduces the amount of bound MTX and increases the free fraction. Decreased osmotic pressure reduces blood volume and causes renal hypoperfusion with reduced glomerular filtration of MTX. Our data showed that pre-infusion serum creatinine values rose with falling serum albumin, supporting the presence of reduced filtration [30]. Low serum albumin increases the fraction of unbound MTX, resulting in greater penetration into “deep” less vascular tissue spaces (V3) that are cleared more slowly. A very low albumin may cause formation of third spaces (pleural effusion, ascites) which may retain MTX for prolonged periods. No such cases were seen in this series.

Renal hypoperfusion seems a plausible explanation for the most striking finding in this study: the impact of the serum albumin level on the steady-state MTX concentration. The median end-of-infusion MTX concentration rose markedly from less than 75 µM in group D to more than 125 µM in groups B and A, and the risk of a very high MTX-h23 ≥ 150 µM tripled from around 10% to more than 30%. This is consistent with reduced MTX elimination depending on the level of serum albumin and corresponds well with the MTX clearances that were determined, falling from an average of close to 11 L/h/1.73 m2 in group D to 9 L/h/1.73 m2 in group A.

A high steady-state MTX concentration is predictive of DME, albeit less predictive than a 50% rise in serum creatinine [8]. We therefore expected to see increased occurrence of creatinine rises and slow elimination in the hypoalbuminemic groups – but none was seen. At 48 h after starting the infusions the differences in serum MTX concentrations had disappeared. The explanation for this accelerated elimination in the hypoalbuminemic groups is not obvious, but prompt institution of hyperhydration 4500 ml/ m2/day with increased alkalinization may have facilitated faster elimination, and faster filtration of the high free fraction may have contributed. It can also be noted that eGFR tended to rise after completing the infusion, especially in groups A and B.

Significant creatinine rises occurred in 10.5% of the courses, oddly with the lowest rates in courses with very low serum albumin. The overall frequency corresponds well with previously reported incidences of 9–18.5% [8, 15, 17, 18, 21, 31]. DME has been defined in various ways in different reports, but it seems clinically reasonable to consider an elimination time > 72 h as prolonged; this occurred in close to 10% of the courses, compared with 17% of 4970 HDMTX treatments in the NOPHO ALL 2000 protocol [32].

While hypoalbuminemia did not delay MTX elimination, the systemic toxicity appearing after the courses was somewhat increased. Oral stomatitis occurred almost twice as frequently in the groups A and B than in groups C and D; a similar difference has been reported in a recent study [22]. The impact on myelotoxicity was less pronounced, with modest increases in low nadir values. The greater toxicity probably stems from increased intracellular accumulation of MTX and formation of polyglutamates during the infusion because of higher free MTX concentrations. It is also in agreement with the higher AUC values, indicating increased MTX exposure.

The clinical consequences of the findings remain to be determined. Strictly, apart from stomatitis the increased MTX concentrations during the infusions had a rather limited effect on the toxicity. It seems prudent, however, to attempt to reduce the risk of reaching a very high MTX concentration during the infusion when serum albumin < 30 g/L. MTX dose reduction would seem rational but may not be necessary: the reduction in clearance and the rise in AUC after all are not very pronounced, GFR appears to become normal during hyperhydration, and MTX elimination is not delayed. An infusion of albumin before starting MTX also seems tempting but may not be recommended because of cost and side effects [16] and probably should be reserved for children with edema, pleural effusion, or ascites. Extension of pre-hydration from 4 to 12 h could be tried; it gave no benefit in a randomized trial [31], but it has been standard practice in Finland and resulted in rapid clearance after 42 or 48 h in 52% of courses [33], compared to 29% in the present series. Hyperhydration with 4500 ml/m2/day from the start of the infusion or increased amount of bicarbonate in the hydration fluid also reasonably could be tried. Measuring serum MTX during the infusion to adjust the infusion rate is another option, but it may not decrease the risk of toxicity [34, 35]. In addition, aiming to avoid stomatitis, an extra folinic acid dose after 36 h or a double dose at 42 h could be considered, and folinic acid mouth rinses could be started early. Under all circumstances, extra care is warranted when starting HDMTX courses with a low serum albumin: fluid balance and urine pH should be monitored meticulously, and nephrotoxic drugs or medications competing for tubular excretion strictly avoided [10].

The impact of hypoalbuminemia on the course of MTX infusions may be overcome, but the relation to ASP therapy raises a question which may have wider repercussions. In current international ALL protocols many HDMTX courses are given while ASP therapy is ongoing. Hypoalbuminemia can be seen as an indicator that ASP has caused effective L-asparagine depletion. The enzyme catalyzes the hydrolysis of L-asparagine to aspartic acid and ammonia, depleting serum and CSF of L-asparagine. Unlike normal cells, lymphoblasts are deficient in asparagine synthetase, and they are therefore “starved” by the depletion, entering resting phase G0-G1 with inhibition of S-phase DNA synthesis [36]. With lymphoblasts forced into metabolic arrest, the effect of antimetabolic drugs like MTX may be antagonized [37]. Thus, it may be speculated that ASP impairs the antileukemic effect of HDMTX while still causing systemic toxicity. It has previously been reported that concomitant administration of ASP has little effect on the toxicity of HDMTX but reduces formation of MTX polyglutamates in cells [38]. Clarification of these issues is of considerable importance.

In conclusion, this descriptive review of HDMTX courses in the treatment of children with ALL has shown that hypoalbuminemia is a frequent occurrence when therapy with ASP is ongoing, and that it has a significant impact on MTX pharmacokinetics, depending on the degree of albumin suppression. We suggest that a level < 30 g/L should be considered clinically significant, defining a group with low initial MTX clearance, high steady-state concentration, high MTX exposure, and doubled risk of stomatitis. Hyperhydration may increase MTX elimination when the end-of-infusion concentration is high, but toxic tissue accumulation may already have occurred, and the utility of an albumin infusion prior to the MTX infusion seems worth investigating. Furthermore, disadvantages from scheduling HDMTX in close proximity to ASP doses merit further deliberation.

## Supplementary Information

Below is the link to the electronic supplementary material.

Supplementary file1 Figure S2. Impact of serum albumin on MTX clearance. Clearances in four groups of HDMTX infusions with different levels of serum albumin (A < 25 g/L, B 25–29 g/L, C 30–35 g/L, D ≥ 35 g/L) are shown. MTX clearance has been divided in three categories: very low (< 8 L/h/1.73m2), low (8–10 L/h/1.73m2), and normal (≥ 10 L/h/1.73m2). Clearance was determined using MTXPK.org (PDF 376 KB).

Supplementary file2 Figure S1. Changes in estimated GFR during and after HDMTX infusions. Averages in four groups of infusions with different base-line levels of serum albumin (A < 25 g/L, B 25–29 g/L, C 30–35 g/L, D ≥ 35 g/L) have been compared. GFR was estimated using the formula eGFR = 36, 5 x (height/serum creatinine) (PDF 379 KB).

## Data Availability

For orginal data, please contact sophie.c@rn.dk.
