# Supplementary figures and images for: Hypoalbuminemia in children with acute lymphoblastic leukemia: relation to asparaginase therapy and impact on high dose methotrexate elimination
Source: Cancer Chemother Pharmacol. 2024 Sep 21;94(6):775–85. doi: 10.1007/s00280-024-04713-0 (PMC11573830; doi:10.1007/s00280-024-04713-0)

Figure S1.

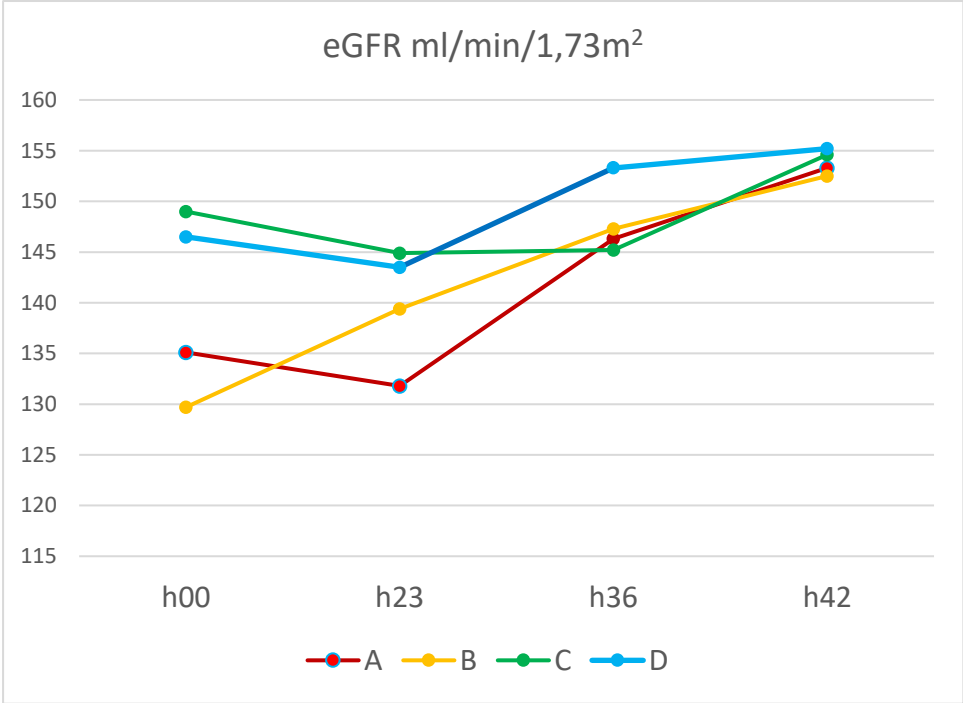

Supplement: Supplementary file 1 — Supplementary file1 Figure S2. Impact of serum albumin on MTX clearance. Clearances in four groups of HDMTX infusions with different levels of serum albumin (A < 25 g/L, B 25–29 g/L, C 30–35 g/L, D ≥ 35 g/L) are shown. MTX clearance has been divided in three categories: very low (< 8 L/h/1.73m2), low (8–10 L/h/1.73m2), and normal (≥ 10 L/h/1.73m2). Clearance was determined using MTXPK.org (PDF 376 KB). [file 280_2024_4713_MOESM1_ESM.pdf]

Figure S2

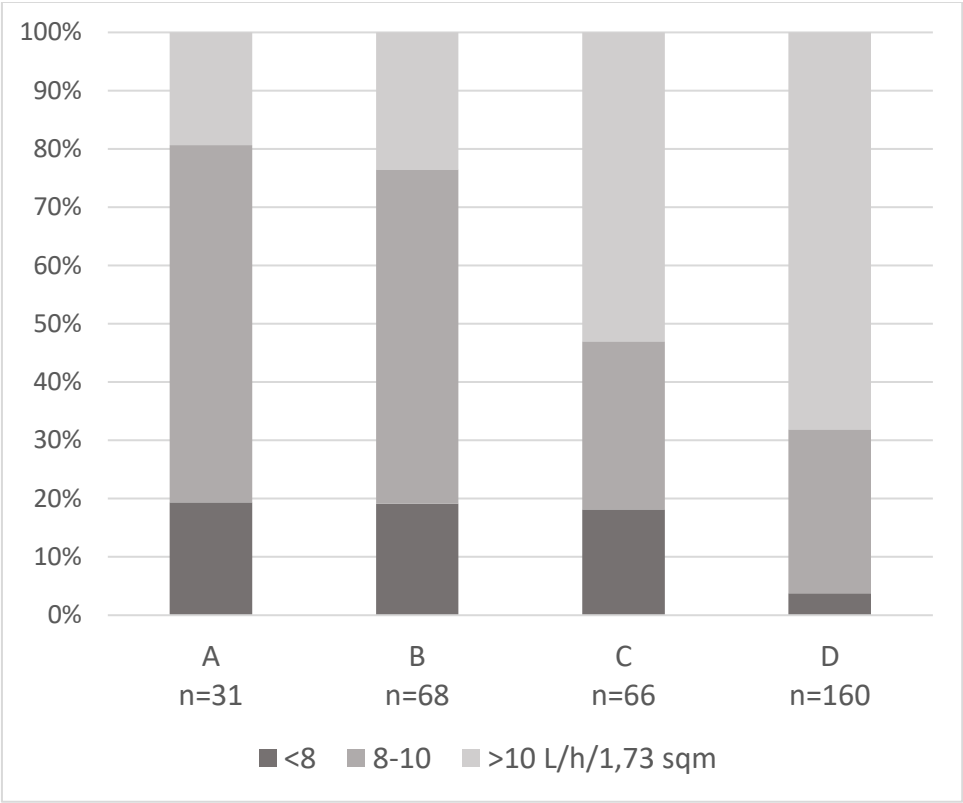

Supplement: Supplementary file 2 — Supplementary file2 Figure S1. Changes in estimated GFR during and after HDMTX infusions. Averages in four groups of infusions with different base-line levels of serum albumin (A < 25 g/L, B 25–29 g/L, C 30–35 g/L, D ≥ 35 g/L) have been compared. GFR was estimated using the formula eGFR = 36, 5 x (height/serum creatinine) (PDF 379 KB). [file 280_2024_4713_MOESM2_ESM.pdf]
